# Genetic testing and immunohistochemistry for SDHB in phaeochromocytoma-paraganglioma syndromes: the South Australian experience

**DOI:** 10.1186/1897-4287-10-S2-A60

**Published:** 2012-04-12

**Authors:** NK Poplawski, L Rawlings, J Seymour, C Vakulin, A Tirimacco, DE Benn, AJ Gill

**Affiliations:** 1 Familial Cancer Unit, Women's and Children's Hospital, North Adelaide, SA, Australia; 2 Familial Cancer Unit, SA Pathology (WCH Site), North Adelaide, SA, Australia; 3 University of Adelaide, Adelaide, SA, Australia; 4 Genetics & Molecular Pathology Directorate, SA Pathology (Frome Road Site), Adelaide, SA, Australia; 5 Cancer Genetics, Kolling Institute of Medical Research, Royal North Shore Hospital, St Leonards, NSW, Australia; 6 Department of Anatomical Pathology, Royal North Shore Hospital, St Leonards, NSW, Australia; 7 University of Sydney, Sydney, NSW, Australia

## Aim and methods

a retrospective review of germline genetic testing (*VHL*, *RET* and succinate dehydrogenase subunit genes *SDHB*, *SDHC* and *SDHD*) and immunohistochemical staining for SDHB in tumour tissue (SDHB-IHC), in patients referred to the South Australian Familial Cancer Unit with an adrenal phaeochromocytoma (PC) and/or paraganglioma (PGL).

## Results

between January 1999 and May 2011, 24 probands were referred to and assessed by our service. The clinical presentation and mutation pick up are presented in the table. Tumour tissue was available from 20 probands and SDHB-IHC was abnormal in all probands with an SDH mutation (5/5; 100%), 0/1 with a *VHL* mutation and 2/12 (16%) with no identified mutation (the 2 probands with abnormal SDHB-IHC both presented with familial head & neck PGL). Tissue was unavailable for testing in the remaining 4 patients; 3 with a *RET* mutation and a MEN2 phenotype; 1 with an *SDHD* mutation and familial head & neck PGL (SDH-IHC is pending in her affected sister). Table 1.

**Table 1:** 

Phenotype	No.	Number with mutation identified					
		Total	*RET*	*VHL*	*SDHB*	*SDHC*	*SDHD*

**Apparently sporadic unilateral PC**	10^	1	-	-	1*	-	-
**Familial PC**	1	1	-	1	-	-	-
**PC & MEN2 features**	3	3	3	-	-	-	-
**Apparently sporadic head & neck PGL**	4	2	-	-	-	1	1
**Familial head & neck PGL**	3	1	-	-	-	-	1
**Apparently sporadic malignant abdominal PGL**	3	2	-	-	2	-	-
**TOTAL**	24	10	3	1	3	1	2

* presented with unilateral PC and developed a head and neck PGL 14 years after the PC

^ *VHL* and *RET* testing incomplete in 2 (complete testing will be presented at the meeting)

## Conclusion

our experience supports using SDHB-IHC as a tool to triage genetic testing in patients with PC or PGL.
